# Personality Functioning and Mentalizing in Patients With Subthreshold or Diagnosed Borderline Personality Disorder: Implications for ICD-11

**DOI:** 10.3389/fpsyt.2021.634332

**Published:** 2021-03-31

**Authors:** Marie Zerafine Rishede, Sophie Juul, Sune Bo, Matthias Gondan, Stine Bjerrum Møeller, Sebastian Simonsen

**Affiliations:** ^1^Stolpegaard Psychotherapy Centre, Mental Health Services in the Capital Region of Denmark, Gentofte, Denmark; ^2^Department of Psychology, University of Copenhagen, Copenhagen, Denmark; ^3^Psychiatric Research Unit, Slagelse, Denmark

**Keywords:** personality disorder, personality functioning, mentalizing, international classification of diseases 11th revision, interpersonal functioning, self-functioning, mediation, borderline personality disorder

## Abstract

The 11th revision of the International Classification of Diseases for Mortality and Morbidity Statistics (ICD-11) defines personality disorder according to personality functioning, which relates to self- and interpersonal functioning. The aim of the present study was to assess the relationship between mentalizing and personality functioning in patients with subthreshold or diagnosed borderline personality disorder. A total of 116 eligible participants were included. Mentalizing was assessed using the Mentalization Questionnaire (MZQ), personality functioning (self- and interpersonal functioning) was assessed using the Level of Personality Functioning Scale-Brief Form 2.0 (LPFS-BF), and borderline severity was assessed using the Zanarini Rating Scale (ZAN-BPD). Mediation analysis was employed to test if mentalizing accounted for the relationship between borderline severity and self- and interpersonal functioning. We found a significant relationship between borderline severity and both subscales of the LPFS-BF. Mentalizing fully and significantly mediated the relationship between borderline severity and interpersonal functioning. However, mentalizing only partly mediated the relationship between borderline severity and self-functioning. Controlling for the covariates gender and age did not impact the results. Mentalizing is likely to be involved in the ICD-11 model of personality functioning, especially interpersonal functioning. This could emphasize the relevance of therapy aimed at strengthening mentalizing abilities when treating personality pathology in general and people with borderline personality disorder in particular. However, self-functioning may be more nuanced, as aspects other than mentalizing also influence self-functioning. The study is explorative in nature and has methodological limitations that require caution in the interpretation and generalizability.

## Introduction

It is estimated that ~8% of the world's population meets the diagnostic criteria for a personality disorder (PD) (1). Besides being a cause of distress to the individual, PDs complicate treatment of somatic and mental disorders, they heighten the risk of morbidity and mortality, and are a large socio-economic burden to society (2). Additionally, PDs are highly prevalent among patients already in the healthcare sector, with ~25% of patients in primary care and 50% of patients in psychiatric outpatient clinics meeting the diagnostic criteria for at least one PD (3).

Historically, there has been a tradition of categorical classification of PDs, such as that followed by all editions of the *International Statistical Classification of Diseases and Related Health Problems* (ICD), published by the World Health Organization (WHO). A definite, categorical classification is sensible as far as the pivotal function of a diagnosis is to assist the clinician in the decision to offer treatment. However, it also has inherent pitfalls that can hamper its clinical utility: the separation into categories lacks reliability, results in high rates of comorbidity, and there remains large clinical heterogeneity within each PD category (4). In addition, the severity of PD, rather than its mere presence, has been shown to influence the course of the disorder and the level of disability that patients experience (5–7). This is partly related to the fact that the threshold for fulfilling a PD diagnosis is arbitrary, which makes the boundary between normal and abnormal personality somewhat artificial (8, 9). In sum, there is no compelling empirical evidence supporting the description of PDs as categorical entities (6, 10–12).

Concurrently, a dimensional classification of PDs has gained momentum, as PDs might be better described by a common pathology factor and specific traits (12–14). Within a dimensional classification, PDs are defined according to severity rather than distinct categories. In the fifth edition of the *Diagnostic and Statistical Manual of Mental Disorders* (DSM-5) (15), the American Psychiatric Association presented the dimensional approach termed *The Alternative Model of Personality Disorder*s (AMPD). This model was placed in a separate section termed *Emerging Measures and Models* (Section III) of the DSM-5, which largely retained the DSM-IV (16, 17) classification. In a bold move, the WHO (18) will abandon the categorical classification altogether in favor of a dimensional approach to PDs in ICD-11, which is to be introduced in all WHO member states in 2022.

The dimensional approach to PDs in both ICD-11 and the AMPD in DSM-5 focuses on the severity of PDs, termed *personality functioning*. Thus, personality functioning is a new term introduced and defined in ICD-11, and though it overlaps with terms like psychosocial functioning, it is not identical (see Table 1 for thorough description). Hence, a PD is assessed by rating its severity on a continuum ranging from normal (healthy) personality functioning through mild and moderate to severe personality pathology. The link between personality pathology and personality functioning has previously been described thoroughly in the literature (19–21) and has been supported empirically (22–25), emphasizing the relevance of impaired personality functioning as a measure of the severity of personality pathology.

**Table 1:** Tentative “Cross Walk” for personality functioning and mentalizing poles.

**Aspects of personality functioning that contribute to severity determination of personality disorder in ICD-11**			**Mentalizing poles**
Degree and pervasiveness of disturbances in functioning of aspects of the self	Stability and coherence of one's sense of identity (e.g., extent to which identity or sense of self is variable and inconsistent or overly rigid and fixed)		Self, automatic, affective/cognitive, internal
	Ability to maintain an overall positive and stable sense of self-worth		Self, automatic, affective, internal
	Accuracy of one's view of one's characteristics, strengths, limitations		Self, cognitive, internal
	Capacity for self-direction (ability to plan, choose, and implement appropriate goals)		Self, cognitive, controlled, internal
Degree and pervasiveness of interpersonal dysfunction across various contexts and relationships (e.g., romantic relationships, school/work, parent-child, family, friendship, peer context)	Interest in engaging in relationships with others		Other, affective, automatic, internal
	Ability to understand and appreciate other's perspective		Other, affective/cognitive, internal
	Ability to develop and maintain close and mutually satisfying relationships		Other, affective
	Ability to manage conflict in relationships		Other, controlled
Pervasiveness, severity, and chronicity of emotional, cognitive, and behavioral manifestations of the personality dysfunction	Emotional manifestations	Range and appropriateness of emotional experience and expression	Self, automatic, affective
		Tendency to be emotionally over- or underreactive	Self, automatic, affective
		Ability to recognize and acknowledge unwanted emotions (e.g., anger, sadness.)	Self, affective, internal
	Cognitive manifestations	Accuracy of situational and interpersonal appraisals, especially under stress	Other, self, cognitive
		Ability to make appropriate decisions in situations of uncertainty	Self, cognitive
		Appropriate stability and flexibility of belief systems	Self, cognitive
	Behavioral manifestations	Flexibility in controlling impulses and modulating behavior based on the situation and consideration of the consequences	Self, cognitive, controlled, external
		Appropriateness of behavioral responses to intense emotions and stressful circumstances (e.g., propensity to self-harm or violence)	Self, controlled, external

*The extent to which the dysfunction on the above areas are associated with distress or impairment in personal, family, social, educational, occupational, or other important areas of functioning*.

In practice, the conceptualizations of personality functioning in the AMPD and ICD-11 share many similarities. For example, both systems involve similar descriptions of self- and interpersonal functioning. Specifically, in ICD-11, the severity of PDs is determined by: “*(1) Impairments in self-functioning, (2) impairments in interpersonal functioning, (3) the pervasiveness, severity, and chronicity of emotional, cognitive, and behavioral manifestations of the personality dysfunction, (4) the extent to which the personality disturbance is associated with distress or significant impairment in personal, family, social, educational, occupational or other important areas of functioning”* (18). Trait domain qualifiers can be added to the diagnosis (e.g., negative affectivity, detachment, dissociality, disinhibition, and anankastia) (18). The ICD-11 working group chose to add a borderline qualifier to facilitate the choice of intervention and to preserve the empirical evidence, which has been gained using the categorical borderline personality disorder (BPD) diagnoses. However, the borderline qualifier is meant to be used only after the level of severity and trait domain qualifiers have been determined (17, 18).

When operationalizing assessment of personality functioning in the DSM-5, Bender and colleagues (26) described that personality functioning is closely linked to the social-cognitive ability of *mentalizing* (27). The concept of mentalizing stems from the psychodynamic tradition and is used to describe the ability to understand and interpret other's and one's own actions in terms of mental states (e.g., feelings, thoughts, and desires) (27). Mentalizing is a multidimensional concept, defined by four dimensions, each with two poles (i.e., self-other, internal-external, automatic-controlled, and cognitive-emotional) (28). When mentalizing is disrupted, three resulting categories of non-mentalizing modes termed *psychic equivalence mode, teleological mode*, and *pretend mode* are automatically activated (29). Mentalizing is closely related to social cognition, metacognition, and reflective functioning, and the terms are often used interchangeably in the literature (30). Thus, in this article, these abilities are referred to as mentalizing. Predictably, mentalizing and aspects of personality functioning have been linked repeatedly in empirical studies (31–35). Similarly, empirical studies have supported the link between mentalizing and personality pathology (36–41), especially BPD (38–41).

Even though mentalizing is not explicitly mentioned in DSM-5 or ICD-11, the close relationship between mentalizing and personality functioning is evident when looking at the aspects of personality functioning that contribute to the severity rating of PDs in ICD-11 (see Table 1 for a tentative crosswalk between aspects of personality functioning and mentalizing poles). However, the association between mentalizing and personality functioning with regard to the ICD-11 model has, to our knowledge, only been empirically investigated in one study (31). The authors used the assessor-rated Reflective Functioning (RF) Scale (42) to assess mentalizing and the Semi-structured Interview for Personality Functioning for DSM-5 (STiP-5.1) (43) to assess personality functioning in clinical and non-clinical samples. They found significant relationships between mentalizing and both self- and interpersonal functioning (31).

In summary, literature suggests that personality pathology, personality functioning, and mentalizing are related concepts. One likely way that these interact is that higher PD severity negatively influences the ability to mentalize. This reduced mentalizing ability leads to reduced functioning in relation to the self as well as others. The theoretical assumption is that PD severity reduces personality functioning, mediated by mentalizing ability.

The aim of the present study was to examine a mediation model in which PD severity acts as the exposure variable, personality functioning as the outcome variable, and mentalizing as the mediator. We examined two mediation models: one with self-functioning and the other with interpersonal functioning as the dependent variable.

We analyzed a sample of patients with subthreshold or diagnosed BPD. Based on the previous research findings, we predicted that: (1) higher BPD severity (i.e., personality pathology) would be linked to lower self-functioning and that this effect would be mediated by mentalizing; and (2) that higher BPD severity would be linked to lower interpersonal functioning and that this effect would also be mediated by mentalizing.

## Methods

### Design

A cross-sectional design was employed with a sample of adult participants with subthreshold (four of nine criteria according to the DSM-5) or diagnosed BPD. We used baseline data from a randomized clinical trial (RCT) assessing the effects of short-term vs. long-term mentalization-based therapy for outpatients with subthreshold or diagnosed BPD (44). At the time the present study was conducted the inclusion of patients to the RCT was ongoing, hence the data used here is from patients recruited from September 2018 to December 2019.

### Sample and Procedure

Participants were recruited from the Outpatient Clinic for Personality Disorders and Trauma at the Stolpegaard Psychotherapy Center, Mental Health Services in the Capital Region of Denmark. Participants were assessed for eligibility using the Mini International Neuropsychiatric Interview (MINI) (45) for general psychopathology, and with the Structural Clinical Interview for DSM-5 Personality Disorders (SCID-5-PD) for personality pathology (46, 47). Participants were eligible for inclusion in the trial if they met the eligibility criteria outlined in Table 2. We chose to include participants with a subthreshold diagnosis because recent empirical research shows that having four out of nine BPD criteria can be equally impairing, similar to a full diagnosis (48).

**Table 2:** Eligibility criteria.

**Criteria exclusive to the outpatient clinic**	**Criteria exclusive for the trial/Study**
- Aged 18–60 - Personality disorder(s) considered to be the primary diagnosis/diagnoses. - Possibility of a learning disability (IQ > 75). - A full diagnosis of antisocial personality disorder or schizotypal personality disorder - Presence of a comorbid psychiatric disorder that warrants specialized treatment - Current (past 2 months) substance dependence including alcohol - Concurrent psychotherapeutic treatment outside the clinic - Unable to speak and understand Danish	- A minimum of four confirmed DSM-5 diagnostic criteria for borderline personality disorder - Written informed consent

The final sample comprised 116 participants. See Table 3 for demographic data. Sixty-four percent of the sample was diagnosed with more than one PD, and 82 percent suffered from other mental disorders as diagnosed using MINI.

**Table 3:** Sociodemographic data.

**Age**	
Mean	32
Range	18–57
**Gender**	
Female	94%
**Ethnicity**	
Danish	91%
Other western	2%
Other	7%
**Civil status**	
Single	50%
Married	8%
In a relationship not cohabiting	19%
Cohabiting with partner	18%
Separated/divorced	5%
**Educational level**	
No educational training	50%
Vocational education and training	5%
Short-cycle higher education	18%
Medium-cycle higher education	23%
Long-cycle higher education	4%
Other	0%
**Job-status**	
Unemployed or at job center	55%
Under education	24%
Self-employed	1%
Unskilled worker	4%
Skilled worker	12%
Stay-at-home	2%
Other	2%

Upon inclusion, participants were interviewed using the Danish version of the Zanarini Rating Scale for Borderline Personality Disorder by trained investigators and filled out the Mentalization Questionnaire and Level of Personality Functioning Scale-Brief Form 2.0.

### Measures

Mentalizing was assessed using the Mentalization Questionnaire (MZQ) (41) which consists of 15 statements that cover different areas of mentalizing: emotional awareness, refusal of self-reflection (teleological mode), psychic equivalence mode, and inability to modulate affect. The MZQ does not capture the last of the most common non-mentalizing modes, that is, the pretend mode. In terms of the mentalizing dimensions, the MZQ covers self-other, cognitive-affective, internal-external, and controlled, but not the automatic pole of mentalizing (49). The developers of the MZQ recommend not using the subscales until they have been further validated, but to resort to the total score (41). Participants rate the degree to which they agree with each statement on a five-point scale. The total score lies between 0 and 60, with high scores indicating good mentalizing abilities. The MZQ has been validated for use with samples with mental disorders but has a poorer ability to detect more sophisticated aspects of mentalizing. The total MZQ score has a test-retest reliability of 0.76 (41).

BPD severity (i.e., personality pathology) was assessed by trained investigators using the Danish version of the Zanarini Rating Scale for Borderline Personality Disorder (ZAN-BPD) (50). ZAN-BPD is a clinician-administered scale assessing each of the nine DSM-5 BPD criteria on an anchored scale from 0 to 4. The rating is based on both the frequency and severity of symptoms in the past 2 weeks. The total score ranges from 0 to 36, with higher scores indicating greater severity (50). The interclass correlation coefficient (ICC) for test-retest reliability has been estimated to be 0.93 (50), and was 0.92 in a random subsample of 40 participants in the RCT study where the data were drawn from (three raters).

Self- and interpersonal functioning was assessed using the Level of Personality Functioning Scale-Brief Form 2.0 (LPFS-BF) (25, 51, 52). The LPFS-BF consists of 12 statements and two subscales covering the domains of self- and interpersonal functioning. Self-functioning is covered by the subscales Identity and Self-direction; interpersonal functioning is covered by the subscales Intimacy and Empathy. Participants rate the degree to which they agree with each statement on a five-point scale. The total score lies between 0 and 36, with high scores indicating low functioning. The LPFS-BF has adequate internal consistency (Cronbach's α = 0.82) for the total scale, and α = 0.79 and 0.71, for the self-functioning and interpersonal functioning scales, respectively (51).

### Ethics and Data Management

Prior to commencing the RCT, ethical approval was obtained from the Regional Research Ethics Committee (ID number H-18023136), and approval for the present study was obtained from the Danish Data Protection Agency (Approval Number: P-2020-732). All participants provided written informed consent before enrollment and were informed that consent could be withdrawn at any point in the study.

### Statistical Analysis

Mediation analysis was performed using the R package lavaan (53). A direct effect was allowed between ZAN-BPD and the outcome of LPFS-BF self- and interpersonal functioning (partial mediation model). In two separate analyses, MZQ scores were defined as the mediator between ZAN-BPD and LPFS-BF/self-functioning or LPFS-BF/interpersonal functioning, respectively. We chose to control for gender and age as covariates in the analysis, as previous research showed that older age (54, 55) and being female (56–58) were both correlated with better mentalizing abilities. We further performed a sensitivity analysis to control for current mental disorders (depression, dysthymia, hypomania, mania, agoraphobia, social anxiety, OCD, PTSD, general anxiety disorder, panic disorder, anorexia and bulimia), as assessed by MINI.

Results were reported as raw regression coefficients along with their 95% confidence intervals (CI), without standardization (which would remove any dimensional information) (59, 60). In line with the exploratory nature of the study, the reported results are based on the per-protocol population, excluding participants with missing data in any of the included variables.

## Results

All participants provided complete data that could be used for the mediation analysis. Overall, a unit increase in ZAN-BPD was associated with an 0.39 increase in LPFS/self-functioning (total effect, 95% CI from 0.23 to 0.55, *p* < 0.001) (a) higher score on LPFS/self-functioning indicates lower functioning). The mediation model divided this effect into a direct effect of 0.24 from ZAN-BPD to LPFS/self-functioning (95% CI 0.10 to 0.38, *p* = 0.001), and an indirect effect of 0.16 (95% CI 0.06 to 0.25 *p* = 0.001), mediated via MZQ. The mediator effect was modest but statistically significant at both stages (*p* < 0.001), with −0.06 (95% CI −0.09−0.03) from ZAN-BPD to MZQ(the negative sign reflects that the rating assesses severity, whereas MZQ assesses the ability to mentalize), and −2.6 (95% CI −3.3−1.9) from MZQ to LPFS/self-functioning (the negative sign reflects that the ZAN-BPD rating assessed severity, MZQ assessed ability to mentalize, and LPFS-BF assessed problems in self-functioning). The influence of the covariates, gender and age, was negligible and not statistically significant.

**Figure 1:**
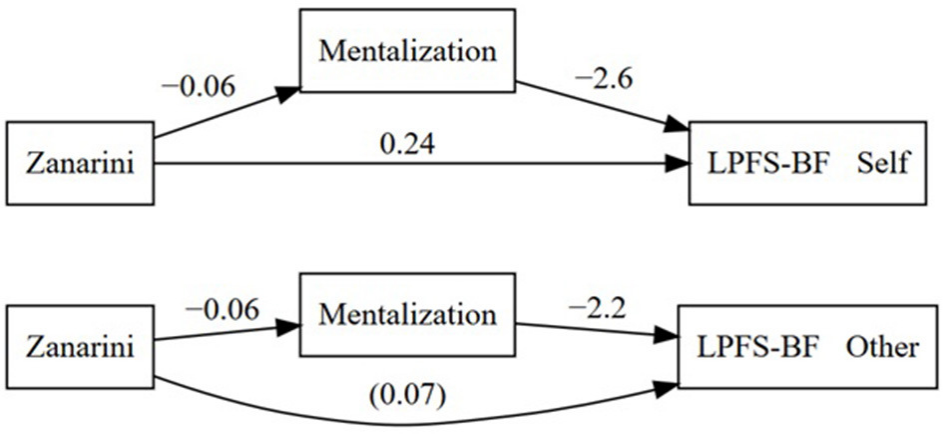
Mediation models for the relationship between BPD severity and self- and other/interpersonal functioning as mediated by mentalizing abilities. The reported coefficients are obtained after controlling for the covariates gender and age.

A unit increase in ZAN-BPD was associated with a 0.20 increase in LPFS/interpersonal functioning (total effect, 95% CI 0.05–0.34, *p* = 0.008). The direct effect from ZAN-BPD to LPFS/interpersonal functioning was not statistically significant, 0.07 (95% CI −0.06–0.20, *p* = 0.34). The mediator effect via MZQ was modest but statistically significant at both stages (*p* < 0.001), with −0.06 from ZAN-BPD to MZQ (see above), and −2.2 (95% CI −2.9−1.5) from MZQ to LPFS/interpersonal functioning (the negative relationship reflects that MZQ assessed ability to mentalize and LPFS-BF assessed problems in interpersonal functioning). The influence of the covariates, gender and age, was negligible and not statistically significant. In other words, for LPFS/interpersonal functioning, a model was supported in which the relationship between ZAN-BPD and LPFS/interpersonal functioning was fully mediated by mentalization. The mediation models are summarized in Figure 1.

## Discussion

The present study examined mediation models of the relationship between BPD severity, mentalizing, and self- and interpersonal functioning. We expected that higher BPD severity would be related to both lower self- and interpersonal functioning. We further expected that mentalizing would mediate this effect.

Our analysis showed a significant relationship between BPD severity, self-functioning, and interpersonal functioning (total effect).

In the first mediation analysis, we found a significant relationship between BPD severity and self-functioning. Mentalizing modestly, but significantly, mediated this relationship (Figure 1). In the second mediation analysis, we found that mentalizing fully and significantly mediated the relationship between BPD severity and interpersonal functioning. We found no significant differences in the results when controlling for age and gender and current mental disorders assessed by MINI, lending no support that these variables moderate mentalizing.

The result that mentalizing fully mediated the relationship between BPD severity and interpersonal functioning confirmed our hypothesis and is in line with prior research on the relationship between both personality functioning and mentalizing (31, 61) as well as personality pathology and personality functioning (22–25). Further, this result is in line with the mentalizing theory according to which mentalizing concerns the apprehension and interpretation of interpersonal interaction and, therefore, has a key function in constructive and meaningful interpersonal functioning (62). When the ability to reflect on the inner states of others and how others experience one's actions is flawed, interpersonal relations are negatively affected (62).

It was unexpected that mentalizing did not fully mediate the relationship between BPD severity and self-functioning, because low mentalizing abilities have been linked to pathology of the self, both theoretically (29) and empirically (31). Our results indicate that the relationship between personality functioning and mentalizing might not suffice in explaining the relationship between personality pathology (i.e., BPD severity) and self-functioning. Accordingly, other aspects likely affect the relationship between personality pathology and self-functioning. One likely contributor might be identity diffusion, which has been described as a core component of personality pathology (63, 64). Identity diffusion is a form of self-fragmentation characterized by problems with self-other boundaries (65), which can lead to loss of commitment to values and goals as well as distress from lack of self-definition and coherence (66, 67). Accordingly, identity diffusion likely influences self-functioning. The link between identity diffusion and self-functioning has been supported empirically (68). However, identity diffusion and mentalizing are likely overlapping concepts. From a mentalizing point of view, identity diffusion arises when mentalizing is impaired. Accordingly, the ability to experience one's own behavior as driven by internal mental states forms a sense of agency and autonomy, which contribute to self-coherence and a sense of the self as separate from others (65). Hence, the operationalization and empirical investigation of mentalizing or identity diffusion as two different aspects contributing to personality functioning might be unachievable.

The lack of full mediation of the relationship between BPD severity and self-functioning could be due to methodological factors. Such factors may include differences in participants' ability to self-report on self- and interpersonal functioning (69) [e.g., those with better mentalizing abilities may be more aware of self-dysfunction and hence report higher levels of dysfunction (70)], as well as shortcomings in the construct validity of the MZQ (e.g., ability to capture automatic mentalizing and pretend mode). Interestingly, some of these methodological issues were already touched upon in the first proposal for the ICD-11 classification, which only defined PDs in terms of interpersonal problems because self-pathology was deemed “too sophisticated to incorporate into a general definition” [(71), p. 250]. However, this position was eventually forsaken because of the need to align the definition of PD with the DSM, but also to pay sufficient attention to a first-person perspective that may have particular benefits for the clinical utility of a classification (72, 73).

The main argument for introducing the ICD-11 definition of PD was to enhance the clinical utility of assigning a PD diagnosis. Consequently, we will devote some space to elaborate on the clinical implications of our results. We acknowledge that the present study is explorative in nature, and more research on this area is needed and so the results and clinical implications should be considered with caution. The finding that mentalizing mediated the relationship between BPD severity and personality functioning, especially with regard to interpersonal functioning, points to the importance of mentalizing as a target of intervention for increasing personality functioning in patients diagnosed with PD (74). This can be facilitated through psychoeducation to the patient regarding this relationship. Additionally, the patient's difficulties and aims regarding mentalizing and personality functioning may be expressed in a mutually understood case formulation. This could stimulate the patient's engagement and motivation to practice their mentalizing ability. However, as mentioned, insight about problematic or inappropriate aspects of personality functioning can be flawed in people with PDs (69, 70), and the development of an agreed-upon case formulation can be challenging. Nevertheless, based on the present results, we find it worthwhile for therapists to engage in this challenging endeavor, as areas of impaired mentalizing about the self and others can be objects of mutual reflection between patient and therapist in therapy. One way the therapist can work with this is to explicitly mentalize their own thoughts and feelings to the patient and simultaneously aid the patient in mentalizing their own thoughts and feelings (63, 75). Hence, one relevant implication of the results is that they lend support for framing PD as a disorder that can be alleviated through the patient's active involvement in psychotherapy.

As mentalizing was initially explicitly mentioned in the description of personality functioning in the initial work on DSM-5 (26), it is unsurprising that we found empirical support for the relationship between personality functioning and mentalizing. However, the term was removed from the official description of personality functioning in DSM-5, because of the risk of being “too unfamiliar or relying excessively on a particular theoretical jargon” [(26), p. 340]. Thus, the results of the present study bring personality functioning back to its initial roots but may also contribute to a more nuanced way of understanding personality functioning with regard to self-functioning.

A strength of the present study is the thorough and structured assessment of the participants' diagnoses. The SCID-5-PD is accepted as the gold standard for the psychiatric diagnosis of PDs (76). Thus, the results are based on a narrowly defined sample of participants, which increases generalizability.

The present study also has some limitations. First, we did not publish a protocol prior to the mediation analysis. Hence, there is a risk of data-driven results. Second, all data were collected cross-sectionally. Therefore, the causal link between BPD severity, mentalizing, and personality functioning is questionable. A longitudinal design could be used to overcome this limitation. However, when the temporal relationship of the constructs measured is not known, it is doubtful that the study will benefit from using a longitudinal design. Often, we cannot be certain about which time lags to choose (77). Choosing arbitrary time points does not provide better evidence than cross-sectional design (77). Third, other risks related to cross-sectional design are common method variance, such as the risk of overestimating correlations because of qualities related to the applied method of enquiry as opposed to actual covariation among the phenomena of interest (78). However, the use of multiple data sources minimized this risk in the present study. Fourth, the test-retest reliability of the total MZQ score has been deemed rather low (0.76) (41). This might be part of the reason that the effect sizes in the current study were rather small. Fifth, there is an inherent paradox in assessing mentalizing through self-report measures since assessing and understanding mental phenomena lies at the very core of mentalizing. Thus, persons with poor mentalizing abilities are likely to have difficulty in precisely reporting their mentalizing skills. However, previous studies have shown that mentalizing can be assessed both reliably and validly through self-reports (41, 79–81). A sixth limitation is the use of the LPFS-BF, which was developed for the DSM-5 AMPD. To this day, there is no official agreement on published instruments on the specific ICD-11 aspects of self- and interpersonal functioning. Accordingly, we chose the LPFS-BF. In terms of face validity, the items also cover the ICD-11 characteristics of self- and interpersonal dysfunction (52). A seventh limitation is that the generalizability of results is possibly hampered because of the skewed gender representation. Finally, we looked at mentalizing as a one-dimensional concept, where mentalizing abilities were rated from poor to better. However, mentalizing is a four-dimensional concept, each of which has two poles (i.e., internal-external, automatic-controlled, affective-cognitive, self-other) (28, 29). Similarly, we did not assess mentalizing deficits such as psychic equivalence, teleological mode, or pretend mode (29). However, to our knowledge, there is no self-report measure that can adequately capture such mentalizing deficits. In light of these limitations the conclusion made here is cautious.

To our knowledge, this is the first study to investigate the link between personality functioning and mentalizing in a PD sample. More research is needed in this area, especially since the transition to ICD-11 is approaching. For example, it would be relevant to assess generalizability by investigating the relationship between self- and interpersonal functioning and mentalizing dimensions in a sample of participants with different forms of personality pathology. It would be relevant to look at the relationship between different forms of personality pathology and mentalizing poles as different forms of personality pathology have been described based on the different mentalizing dimensions (82); however, this still lacks empirical support. Additionally, it would be valuable to insert both mentalizing and identity diffusion in a similar mediation model as the one assessed in the present study. Finally, it would be relevant to investigate whether a clinical intervention aimed at enhancing mentalizing would result in improved personality functioning, as the present results suggest.

## Data Availability Statement

The raw data supporting the conclusions of this article will be made available by the authors, without undue reservation.

## Ethics Statement

The studies involving human participants were reviewed and approved by the regional research ethics committee of Mental Health Services in the Capital Region of Denmark. The patients/participants provided their written informed consent to participate in this study.

## Author Contributions

The initial idea of investigating personality pathology, mentalizing, and personality functioning in a mediation analysis was conceived by MR, SJ, SB, and SS. MR was the main author of this article. SJ, SB, MG, SBM, and SS contributed to writing, discussing, and supervising the process and content of the article. MG designed the plan for statistical analyses and wrote the sections on the statistical approach and results. All authors read and approved the final manuscript.

## Conflict of Interest

The authors declare that the research was conducted in the absence of any commercial or financial relationships that could be construed as a potential conflict of interest.
