# Supplementary material for: Personality Functioning and Mentalizing in Patients With Subthreshold or Diagnosed Borderline Personality Disorder: Implications for ICD-11
Source: Front Psychiatry. 2021 Mar 31;12:634332. doi: 10.3389/fpsyt.2021.634332 (PMC8044580; doi:10.3389/fpsyt.2021.634332)
Supplement: Supplementary file 1 [file Data_Sheet_1.PDF]

Appendix

In a sensitivity analysis we further controlled for the influence of other current mental disorders. Here, a unit increase in ZAN-BPD was associated with a 0.35 increase in LPFS/self-functioning (total effect, 95% CI from 0.19 to 0.51, $p < .001$) (a higher score on LPFS/self-functioning indicates lower functioning). The mediation model divided this effect into a direct effect of 0.22 from ZAN-BPD to LPFS/self-functioning (95% CI from 0.07 to 0.58, $p = .002$), and an indirect effect of 0.13 (95% CI 0.04 to 0.23 $p = .006$), mediated via MZQ. The mediator effect was modest and not statistically significant at both stages, with -0.05 (95% CI -0.08 to -0.02, $p = .003$) from ZAN-BPD to MZQ (the negative sign reflects that the rating assesses severity, whereas MZQ assesses the ability to mentalize), and -2.5 (95% CI -3.3 to -1.9, $p < .001$) from MZQ to LPFS/self-functioning (the negative sign reflects that the ZAN-BPD rating assessed severity, MZQ assessed ability to mentalize, and LPFS-BF assessed problems in self-functioning). The influence of the covariates, gender, age, and other current mental disorders, was not statistically significant.

In the sensitivity analysis for interpersonal functioning a unit increase in ZAN-BPD was associated with a 0.20 increase in LPFS/interpersonal functioning (total effect, 95% CI 0.05 to 0.35, $p = .011$). The direct effect from ZAN-BPD to LPFS/interpersonal functioning was not statistically significant, 0.08 (95% CI −0.06 to 0.22, $p = .24$). The mediator effect via MZQ was modest and not statistically significant at both stages, with -0.05 from ZAN-BPD to MZQ (see above), and -2.2 (95% CI -2.9 to -1.5, $p < .001$) from MZQ to LPFS/interpersonal functioning (the negative relationship reflects that MZQ assessed ability to mentalize and LPFS-BF assessed problems in interpersonal functioning). The influence of the covariates, gender, age, and other current mental disorders was not statistically significant.
